# The Influence of External Breast Prostheses on the Body Postures of Women Who Have Undergone Mastectomies

**DOI:** 10.3390/jcm12072745

**Published:** 2023-04-06

**Authors:** Anna Koralewska, Małgorzata Domagalska-Szopa, Jan Siwiec, Andrzej Szopa

**Affiliations:** 1 Department of Developmental Age Physiotherapy, Medical University of Silesia in Katowice, 40-751 Katowice, Poland; 2 John Paul II Pediatric Center in Sosnowiec, 41-218 Sosnowiec, Poland; 3 Department of Physiotherapy, Medical University of Silesia in Katowice, 40-751 Katowice, Poland

**Keywords:** body posture, external breast prosthesis, mastectomy, moiré topography, breast cancer

## Abstract

Most women who have had a mastectomy and have not opted for breast reconstruction choose to use an external breast prosthesis. This study aimed to assess the impacts of external breast prostheses on the body postures of women after unilateral mastectomies. An additional aim was to identify whether postural asymmetry depended on the side of mastectomy. This study involved 52 women after unilateral mastectomy and consisted of two parts: (1) anthropometric measurement and (2) assessment of body posture using the moiré topography method. The posturometric indices showed that the body posture of the subjects in the sagittal plane is characterized by forward trunk inclination and a tendency to excessive kyphosis. There were no significant differences between parameters characterizing body posture with and without external breast prosthesis. The lack of external breast prosthesis had a significant effect only on excessive forward trunk inclination. Significant differences were found in the posturometric parameters in the transverse plane between the groups of patients after left- and right-sided mastectomy. The obtained results did not fully confirm the hypothesis that the external breast prosthesis affects the body posture of women after unilateral mastectomy.

## 1. Introduction

Breast cancer is the most common type of cancer worldwide across the female population [1] and the most prevalent cancer-related cause of death among women [2]. Breast cancer has become a significant public health problem, as both the incidence and mortality rates of the disease continue to rise annually [3].

One of the main treatments for breast cancer is surgery. Although there has been a recent trend towards breast-conserving surgery, a mastectomy is often needed. Mastectomy involves the amputation of the entire breast, often combined with removing lymph nodes. Sometimes there is also a need to remove the fascia of the pectoralis major and pectoralis minor [4,5]. In addition to the surgical procedure, appropriate complementary therapy is also selected to consolidate the radical nature of the treatment. The most common postoperative complications after mastectomy include infection, seroma, hematoma, postoperative pain, tissue necrosis, wound dehiscence, thromboembolism, sensory disturbances, superficial thrombophlebitis, lymphoedema, limited mobility in the shoulder joint, and nerve damage [6].

Among the recently discussed breast cancer issues is the postural control assessment in women after mastectomy [7,8,9,10,11,12,13,14,15,16,17,18,19,20,21,22,23,24,25,26,27,28,29,30,31,32,33,34,35,36,37,38,39,40,41,42,43,44,45,46,47,48]. A review of the above-mentioned publications indicated that the undertaken research was concerned with the impact of mastectomy on body static conditions [7,8,9,10,11,12,13,14,15,16,17] and body posture of women who had received mastectomies [7,10,18,19,20,21,22,23,24,25,26,27,28,29,30,31,32,33,34,35,36,37,38,39,40,41,42,43,44,45,46,47,48] and also the impact of external breast prostheses on postural control in women after mastectomies [11,12,14,32]. The results of several scientific reports indicate that mastectomies may cause body posture disorders such as asymmetry in the trunk in the frontal plane [18,21,42,43,44,45,46,47], asymmetry in the shoulder girdle [23,42,43,44,45,46], the curvature of the spine [18,30,34,48], anterior–posterior [10,19,24,27,41] deepening of thoracic kyphosis [11,23,34,41,47], deepening of lumbar lordosis [47], and pelvic abnormalities [10]. Researchers are currently trying to answer questions surrounding whether the body postures of women after mastectomy depend on which side of the body was operated on [7,19,20,32,34,47], the type of procedure performed [20,21,24,34,39], lymphedema [26,27,31], the time elapsed since the procedure [21], breast reconstruction [22,25,28,30,33,40], and type reconstruction [25] as well as the type of physical activity practiced or rehabilitation [19,35,36,37,38]. The literature on the subject and popular opinion have indicated the importance of external breast prostheses in restoring correct trunk statics and protecting against body posture disorders and balance disorders [32]. However, some studies do not confirm the significant impact of external breast prostheses on the occurrence of postural control disorders [11,12,16,32]. Such controversies have encouraged the authors to recognize the problem of postural control disorders in women after unilateral mastectomies as part of a broader research project, “Postural control disorders in women after unilateral mastectomy.” The results of the first part of this project, consisting of comparing the measurement of weight-bearing distribution between the amputated and non-amputated side of the body in conditions with and without an external breast prosthesis, showed that: (1) the occurrence of lymphoedema in the upper limb on the amputated side did not affect the nature of the asymmetry in weight-bearing distribution between the amputated and non-amputated side of the body [16] and (2) the external breast prosthesis does not have a major impact on the postural stabilities of women after unilateral mastectomy [12]. The obtained results encouraged us to extend the scope of our research by assessing the impacts of external breast prostheses on the body postures of women following unilateral mastectomy. Although results in the literature show that unilateral mastectomy causes body posture disorders [7,10,18,19,20,21,22,23,24,26,28,33,34,39,41,42,43,44,45,46,47], a small number of reports do not confirm the influences of external breast prostheses [32].

As part of this study, an attempt was made to assess the impacts of external breast prostheses on the body postures of women after unilateral mastectomies by comparing the results of moiré topography (MT) assessments of their bodies in free-standing positions performed with vs. without external breast prostheses. First, it has been hypothesized that women who undergo unilateral mastectomy experience body posture disturbances, mainly in body faults in the frontal plane. Second, the external breast prostheses (EBP) significantly counter postural asymmetry in this population. An additional aim of this study was to identify whether postural asymmetry in women who underwent unilateral mastectomy depended on the side of mastectomy.

## 2. Material and Methods

### 2.1. Study Participants

This study involved 52 women who underwent unilateral Modified Radical Mastectomy or Simple Mastectomy. They were participants in the European Union’s “You are worth it” program, organized by the Sanus Medical Clinic in Zabrze, Poland. This program aimed to provide comprehensive care and rehabilitation for a group of women after mastectomy. This was the same group of women who had participated in our previous study on the “Postural Control of Women Who Underwent Mastectomy” [12]. The mean age of the participants was 61.8 ± 10.8 years. The participants’ average body weight was 78.40 kg, total body length was 160.02 cm, body mass index (BMI) was 30.7, and the mean time since surgery was 6.5 ± 7.6 years. Twenty-seven study participants had a left-sided mastectomy, and twenty-five had a right-sided one. In all the women, the right upper limb was the dominant upper limb. All participants had a history of combined treatment, i.e., total or modified mastectomy, chemotherapy, and/or radiotherapy. All respondents declared that they wore external breast prostheses, which they used at least during the day. The prostheses used by the patients were selected by a trained person. Patients were included in this study based on the inclusion and exclusion criteria. The inclusion criteria for this study were as follows: (1) female sex, (2) history of unilateral total or modified mastectomy, (3) use of an external breast prosthesis at least during the day for at least six months, and (4) written declaration of informed consent to participate in the study.

Exclusion criteria included: (1) diagnosed vertigo, (2) diagnosed diseases of the nervous system, such as stroke, Parkinson’s disease, peripheral nerve damage, and paralysis, (3) use of drugs that may affect balance, (4) balance disorders, and (5) diagnosed disorders of the skeletal system such as posture defects or foot deformities, condition after injuries, diagnosed rheumatic diseases, metastases to the skeletal system, and mental disorders such as depression or dementia.

Before the start of this study, all participants were informed about the purpose and individual components of the study and that the participation was voluntary. This research project received the approval of the Bioethics Committee of the Medical University of Silesia in Katowice (Resolution No. KNW/0022/KB1/61/18). This study was conducted in accordance with the Declaration of Helsinki.

The examination consisted of two parts: (1) anthropometric measurements (measurements of body height, weight, and length of lower limbs) and (2) assessment of body posture using the moiré topography method.

### 2.2. Anthropometric Measurements

A scale with a height gauge was used to measure the height and weight of the study participant.

The length of the lower limbs was measured using a tailor’s tape resistant to stretching. The distance between the greater trochanter of the femur and the medial malleolus was measured separately for both lower limbs.

### 2.3. Assessment of Body Posture

The moiré topography method was used to assess the participants’ body posture. This type of optical surface topography method allows for the assessment of the back surface that can be used due to the existence of a relationship between the deformation of the human skeleton and the deformation of the trunk surface. The moiré topography method is a non-invasive method that allows for a quick but measurable three-dimensional assessment of body posture while giving the opportunity to collect, store, and analyze the obtained data [19,33].

Using the moiré topography method, the body posture of the participants in a free-standing position was assessed twice, first with an external breast prosthesis and then after its removal. Each time, the spatial arrangement of individual body segments, such as the shoulder and pelvic girdles and the spine, was recorded in all three planes, i.e., in the sagittal, frontal, and transverse planes.

The examination was carried out using the KBPC Mora 4G body posture photogrammetric assessment kit manufactured by CQ Electronic System, Artur Świerc (Poland), with its accompanying proprietary software, in accordance with the manufacturer’s instructions. The projection-receiving device was connected to a computer and placed on a tripod at a distance of 2.6 m from the examined person. The examination was carried out in a darkened room.

Prior to the start of the examination, the following anthropometric points were marked on the participant’s back with a passive marker: external occipital protuberance, C7 spinous process, line of spinous processes of the vertebrae, inferior angles of the scapulae, superior iliac spines, peaks of thoracic kyphosis and lumbar lordosis, and the thoracolumbar junction [33].

**Figure 1:**
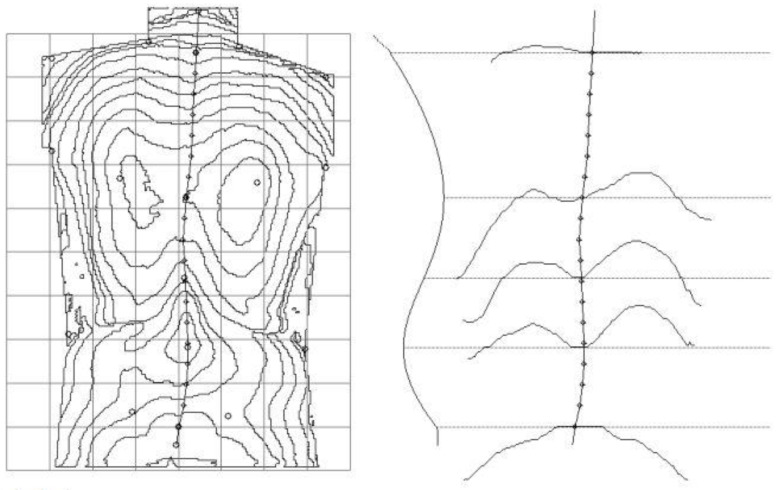
MT examination of a patient after unilateral mastectomy—topography of the back surface, the shape of the spine in the sagittal plane, and cross-sections of the spine at the following heights: vertebra C7, the apex of thoracic kyphosis, thoracolumbar junction, the apex of lumbar lordosis, and vertebra S1 (own elaboration).

Based on the marked anthropometric points, the basic postural indicators were automatically calculated (Table 1, Figure 1).

### 2.4. Statistical Analysis

Statistical analysis was performed using Statistica 13.1 (TIBCO Software Inc., Palo Alto, CA, USA). In order to recognize the differences in posturometric parameters, the indicators in the frontal and transverse planes from the left and right sides to the amputated and non-amputated sides were transformed. Descriptive statistics were calculated for the examined variables, and the Grubbs test was performed. The Shapiro–Wilk test was used to determine the normality of the data distribution. The differences in the values of postural indices with and without external breast prostheses were determined using the Student’s *t*-test or the Wilcoxon test. The Student’s *t*-test was used for statistical calculations if the examined variables followed a normal distribution. The Wilcoxon test was used for non-normally distributed variables. The Student’s *t*-test or the Mann–Whitney U test was used to identify differences in postural indices (results of photogrammetric examinations) between the amputated sides of the body. If the variables had a normal distribution, the Student’s *t*-test was used; if the variables had a non-normal distribution, the Mann–Whitney U test was used. Statistical significance was considered at *p* < 0.05.

## 3. Results

The values of the analyzed postural indicators are summarized in Table 2. The values of the postural indicators describing the body postures of women after unilateral mastectomy do not reflect the actual postural features. As has been indicated in the statistical analysis, the values of these indicators, apart from the value of their deviation from the body axis in a specific plane, reflect the direction of deviation from the body axis. The values with the “–” sign indicate anterior and non-amputated deviations, while “+” signed values indicate posterior and amputated deviations. This does not apply to the indices describing the angles of spinal curvatures (thoracic kyphosis angle—TKA, lumbar lordosis angle—LLA, and compensation index—CI) and the angles of lateral curvatures in the spine (angle of lateral curvature on the amputated side—LCA and angle of lateral curvature on the non-amputated side—LCNA). The values given in Table 2 reflect the actual average values of these parameters.

The values of the posturometric indices obtained as part of the descriptive statistics showed that the body postures of the participants in the sagittal plane were characterized by forward-tilting of the trunk (negative mean torso leaning angle—TLA values) with a tendency to kyphosis (there were relationships between the depth of thoracic kyphosis and lumbar lordosis; TKA, LLA, and CI) (Table 2).

The analysis of posturometric indicators in the frontal plane showed that all participants had a slight lateral curvature in the spine. The angular values of these curvatures did not indicate any disturbances in the symmetry of the spinal position. The latter has concluded the fact that the mean value of the angle of curvature in the spine, which was directed either to the amputated side (LCA) or the non-amputated side (LCNA), was ca. 5°. The highest recorder value was 13° (Table 2). Interestingly, for slightly more than half of the participants, the curvature was toward the amputated side (n = 27; 52%), and in the other half (n = 25; 48%), it was toward the non-amputated side.

The analysis of the posturometric indices showed that, although the average values of the postural indices in the transverse plane were close to 0.00 and did not directly indicate excessive trunk rotation at the level of the scapula (TRA) and pelvis (PRA), the values of the standard deviations exceeded the average values several times, and the maximum values of rotation in both directions were several degrees.

The direction of rotation analysis for TRA and PRA indicated that, among the majority of the participants, both girdles were twisted toward the amputated side. The average angle of rotation in TRA and PRA were subsequently 4.5° (n = 32; 62%) and 4.6° (n = 31; 60%). In the remaining group of participants, TRA and PRA were twisted toward the non-amputated side. The rotation angles of TRA and PRA were subsequently 4.6° (n = 20; 38%) and 6.2° (n = 21; 40%).

While assessing the impacts of external breast prostheses on the body postures of the participants, the average values of the postural indices were compared between the test with and without the external breast prosthesis. There were no statistically significant differences in the posturometric parameters characterizing body posture between the conditions with and without the external breast prosthesis (Table 3). The only statistically significant difference was in the trunk leaning angle (TLA). Even though the average values of TLA were negative in both conditions, indicating that, the trunk was tilted in relation to the axis in the anterior direction (TLA <0.00), the forward tilt in the trunk was significantly greater in the scenario without the external breast prosthesis (Table 3).

In accordance with the other objective of the study, which was to identify differences in postural parameters between patients after right- and left-sided mastectomies, a separate statistical analysis of the individual groups and between them was carried out. The average values of posturometric indicators describing the body posture in the sagittal and frontal planes in the individual subgroups were similar and showed consistent tendencies with those found in the overall study group (Table 4).

However, the analysis of subgroups revealed statistically significant differences in the parameters describing body posture in the transverse plane, i.e., the pelvic rotation angle (PRA) and the trunk rotation angle at the level of the scapula (TRA), depending on the side of the mastectomy. Moreover, these differences were found in both study conditions—with and without external breast prostheses (Table 4).

The mean pelvic rotation angle (PRA) values within the group of left-sided amputees were positive, reflecting rotation in the pelvis toward the amputated side (i.e., anteversion in the iliac crest of the pelvis on the anteriorly non-amputated side). These values remain positive regardless of external breast prosthesis usage. The average values of the trunk rotation angle at the scapula (TRA) level in patients after left-sided mastectomy in the trials with and without the external breast prosthesis were positive, which meant that the trunk rotated toward the amputated side. In contrast, within the group of right-sided amputees, the average values of TRA and PRA were negative, which meant that the pelvis and the trunk rotated in the opposite direction to the amputated side. These values remain negative regardless of external breast prosthesis usage. No statistical significance was found for the remaining postural indicators (Table 4).

## 4. Discussion

Moiré topography is a method that allows for the precise analysis of the locations of individual body segments (e.g., spine, shoulder girdle, and hip girdle) in relation to the body’s main axis in three planes. Because of its high accuracy, MT has been widely recognized as a gold-standard research tool to study the body postures of women who have had mastectomies [19,20,21,23,24,29,33,35,36,38,39]. The results of the above studies report the occurrence of body posture disorders in women following mastectomies, particularly in the sagittal plane [10,19,24,27]. Rostkowska et al. observed that women with mastectomies tended to lean forward compared to non-mastectomized controls [19]. Malicka et al., comparing the body postures of women after mastectomies to their non-mastectomized counterparts, showed that after the procedure, there were tendencies toward kyphosis of the trunk (i.e., deepening of thoracic kyphosis and forward-inclination of the trunk) [41].

Głowacka et al. compared women’s postures following radical mastectomies (the Patey method), breast-conserving surgeries, and controls with no surgical interventions. They found that the postures of women who underwent radical mastectomies were significantly more forward-leaning than those of controls or those of women who underwent breast-conserving surgeries [24]. Mangone et al. compared women who were using post-mastectomy external breast prostheses or tissue expanders with controls who had not had surgical interventions. They observed a significant difference in the anteroposterior trunk flexion in patients with mastectomies [10]. The anterior–posterior trunk flexion in post-mastectomy patients was significantly greater than among the controls. Haddad et al. [27] observed excessive forward bending in the trunk among post-mastectomy women with lymphedema and post-mastectomy women without lymphoedema.

Although the primary objective of this project was not to assess body posture disorders in women after mastectomies, the results of the indicators describing their body postures in the sagittal plane (both with and without external breast prostheses) seem to confirm the above-quoted findings. This is confirmed by both negative mean TLA values exceeding 10 degrees and significant mean values of the depth of thoracic kyphosis and by positive values of the CI, indicating the predominance of thoracic kyphosis over the depth of lumbar lordosis. At this point, however, it should be noted that both the criterion of correct posture in the sagittal plane and body posture defects in this plane are not clearly defined; thus, conclusions about postural disorders resulting from the comparisons of different populations (e.g., mastectomized/non-mastectomized) should be taken with caution. The body posture in the sagittal plane is considered to be correct if the vertical projection of the external auditory canal is projected in front of the shoulder, hip, knee, and ankle joints and runs parallel to the long axis of the body [49]. Describing the body postures of women after mastectomies in these categories, our results, as well as the previous results of other studies, indicate that a forward tilt in the trunk and a tendency toward excessive thoracic kyphosis are some of the defining characteristics of women who have had mastectomies [10,19,24,27]. It has also been acknowledged that large-volume breasts cause a deepening in the angle of thoracic kyphosis and lumbar lordosis [50,51].

Our study found a slight asymmetry in the positioning of the shoulder blades, spines, and anterior superior iliac spines in all examined women, regardless of whether they had a prosthesis or not. The directions of these deviations have not depended on the side of the mastectomy and have been confirmed by the almost proportional distribution of participants in whom the shoulder girdle, pelvic girdle, and spine were oriented once to the amputated side and once to the opposite side. The average angular values of deviations in the frontal plane oscillated in the range of 0.08–1.75, and the maximum values were close to the measurement error (5°).

Our observations are partially confirmed with the X-ray images study conducted by Serel et al. based on an analysis of spine and shoulder positions before and 12 months after unilateral mastectomies, where they noticed a deepening in the lateral angle of the spine in most patients. As in our results, the increase in the angle of lateral curvature in the spine was not dependent on the side of the amputation [18]. The occurrence of torso asymmetry, with particular emphasis on the shoulders and shoulder blades after mastectomy, was reported in photogrammetric studies as early as in the 1990s by Dobosz et al. [42,43]; in recent years, the results were also confirmed by Bąk and Cieśla Drzał-Grabiec et al. and Rangel et al. [19,21,44,45]. In turn, the research results using the MT method conducted by Rostkowska et al. on women after mastectomy vs. non-mastectomized women confirmed the presence of significantly higher values of thoracic kyphosis and lumbar lordosis after mastectomy. Similar to the findings of our study, no differences in the shaping of postures in the frontal plane were found between the studied groups [19].

Assuming the concentricity of the shoulder girdle and pelvis as the criterion of correct body posture in the horizontal plane, postural disorders in this plane, not reported in previous studies, were diagnosed in both examined conditions (with and without the prosthesis). Although the average rotation values of both the shoulder girdle and the pelvis oscillated around zero, the standard deviation values recorded here exceeded the average values of these parameters several times. The maximum values of rotation in both directions reached several degrees. In addition, rotation of the shoulder girdle and pelvis towards the amputated side (i.e., anterior rotation of the scapula/pelvis on the non-amputated side) was more common (62% and 60% of the participants) than in the opposite direction.

The significance of the amputation side on the body posture was particularly interesting. Grouping the participants by left- or right-sided amputation (Lmas; Rmas) revealed new observations. Results showed that the parameters describing body posture in the horizontal plane, i.e., trunk rotation angle at the level of the scapula and pelvic rotation angle (in both conditions), were significantly higher in the participants who had left-sided mastectomies than in those who had right-sided ones. In the Lmas participants, the trunk and pelvis twisted toward the amputated side (i.e., ante-torsion of the scapula/pelvic iliac plate on the non-amputated side toward the front), while in Rmas participants, the rotation of the trunk and pelvis occurred in the opposite direction to the amputation. Similar differences in posturometric indices between Lmas and Rmas were previously reported by Barbosa et al. and Kabała et al. [20,47]. Barbosa et al. compared the body postures of women who had unilateral mastectomies with those of women who had quadrantectomies. They noted that in women who had undergone amputation of the left breast, protraction of the shoulder and inclination of the trunk towards the operated side occurred soon afterwards [20]. Kabala et al. noted a greater angle of lateral curvature in the spine, a greater angle of lateral deviation in the trunk, and general trunk rotation in patients after amputation of the left breast [47].

Although it is difficult for us to justify the differences between left- and right-sided mastectomies, this should not obscure the key finding of our research, which is the diagnosis of the shoulder girdle and pelvis positioning disorders in the horizontal plane after mastectomy. An earlier study by Atanes Mendes Peres et al. [22], which compared the body postures of women who had mastectomies alone with those of women who had mastectomies with simultaneous breast reconstruction, was a harbinger of spinal rotation disorders after mastectomy. A clear tendency toward trunk rotation was observed in women with mastectomies without reconstruction of the removed breast. The research tool that limited the body posture assessment in the transverse plane did not allow for a detailed diagnosis of these disorders [22].

The main objective of this study was to assess the impacts of external breast prostheses on the body postures of women who had unilateral mastectomies. For this purpose, the results of MT assessments in free-standing positions with and without external breast prostheses were compared. The results obtained in this study have not indicated the impacts of external breast prostheses on the body postures of these women. Statistical analysis did not show significant differences in almost all postural indicators between the participants with external breast prostheses and those without. Our results did, however, allow us to observe that the trunk leaning angle in the forward direction was significantly greater in the assessment of body posture without an external breast prosthesis. Considering that it is the most frequently indicated body posture disorder in women who have had mastectomies, the obtained results clearly indicate an impact of external breast prostheses on the trunk leaning angle.

In this study, the results were not compared to those of non-mastectomized controls. Instead, a unique approach was used, which was to compare the posture of the same participant in two different postural conditions; hence, comparing the current results with the results of other studies is difficult. The only prior report on the impacts of external breast prostheses on the body postures of women following mastectomies is the work of Hojan et al. [32]. Although it differs in both purpose and design, some of their results are consistent with ours. The aim of Hojan et al.’s study was to determine whether the weight of the external breast prosthesis may contribute to changes in body posture in mastectomized women. Body posture was assessed based on the activity of the spinal extensor muscle, the activity of which was measured using surface electromyography. The study was conducted under four conditions—without external breast prostheses and with three different weights of external prostheses. The results showed that the presence of an external breast prosthesis did not affect the activity of the erector spinae muscle and did not affect body posture, which is consistent with our results. Perhaps this is because the weight of the female breast (only 4.4% of the total adipose tissue mass) in relation to the total body weight is not as biomechanically significant as it is commonly believed to be [32]. In addition, as mentioned in the Introduction, the results from the first part of our project, consisting of comparing the measurements of weight-bearing distribution between the amputated and non-amputated side of the body in conditions with and without an external breast prosthesis, did not confirm a significant impact of the external breast prosthesis on the postural stabilities of women who had unilateral mastectomies [12].

### 4.1. Clinical Implications

The commonly recognized goal of post-mastectomy physiotherapy is analgesic and anti-swelling physiotherapy and obtaining a full range of motion within the upper limb on the amputated side. This study confirmed the tendency to tilt the trunk forward in women after unilateral mastectomy and indicated that shoulder and pelvic axial alignment disturbances contribute to a better understanding of the biomechanical consequences of mastectomy, which may justify the need to extend both early physiotherapeutic intervention and long-term physiotherapy in mastectomy patients.

### 4.2. Study Limitations

Our study has some limitations including a diverse population of participants in terms of adjuvant treatment methods, which were not taken into account. Although, in terms of the varied age and time since mastectomy of the study population, this study is consistent with earlier ones that addressed this problem, the too-wide age range of the respondents and the time since mastectomy could be the reason that the trunk symmetry disorders in the frontal plane reported in earlier studies were not obvious. These limitations will be taken into account in further stages of our research.

## 5. Conclusions

The results of this study confirmed those of earlier reports that an excessive forward inclination in the torso and tendencies toward kyphotization of the torso characterizes the body postures of women who have undergone mastectomies. Our research allowed us to analyze patterns of axial positioning in the shoulder girdle and pelvis, which indicated that in participants who had received left-sided mastectomies, the shoulder girdle and pelvis twisted toward the amputated side, while in participants who had right-sided mastectomies, there was twisting in the opposite direction to the amputation. The results obtained in this study did not fully confirm the hypothesis put forward at the beginning that external breast prostheses affect the body postures of women who have undergone unilateral mastectomies. However, the absence of an external breast prosthesis during the assessment of body posture significantly impacted the deepening in the angle of forward inclination in the trunk in our study population. Since they are commonly indicated in the literature on the participant of body posture disorder following mastectomy, the positive effects of external breast prostheses on alleviating this disorder should be considered.

## Figures and Tables

**Table 1:** Characteristics of the examined postural indicators in the sagittal, frontal, and transverse planes.

Postural Indicators	Definition
Postural Indicators in the Sagittal Plane	
trunk leaning angle (TLA)	In the sagittal plane, the angle between the line connecting the spinous processes C7-S1 and the vertical. Negative values indicate a forward tilt of the torso, and positive values indicate a backward tilt of the torso.
thoracic kyphosis angle (TKA)	The angle that completes 180° for the angle between the line that connects the C7 spinous process and the spinous process at the top of the thoracic kyphosis and the line that connects the spinous process at the top of the thoracic kyphosis and the spinous process at the level of the transition thoracolumbar. Negative values indicate lordosis, and positive values indicate kyphosis.
lumbar lordosis angle (LLA)	The angle that completes 180° for the angle between the line that connects the spinous process at the level of the thoracolumbar junction and the spinous process at the top of the lumbar lordosis and the line that connects the spinous process at the top of the lumbar lordosis and spinous process S1. Negative values indicate kyphosis, and positive values indicate lordosis.
compensation index (CI)	Parameter resulting from the difference between the angle of thoracic kyphosis and the angle of lumbar lordosis.
Postural Indicators in the Frontal Plane	
trunk inclination angle (TIA)	In the frontal plane, the angle between the line connecting the spinous processes C7-S1 and the vertical. Negative values indicate the inclination of the trunk in the opposite direction to the amputation, while positive values indicate the inclination of the trunk towards the amputation.
pelvic inclination angle (PIA)	The angle between the line connecting the posterior superior iliac spines and the horizontal. If the values are positive, then the pelvis is tilted towards the amputation. On the other hand, if the values are negative, then the pelvis is tilted in the opposite direction to the amputation.
inclination angle of the shoulder line (SLA)	The angle between the line that connects the shoulder processes of the shoulder blades and the horizontal. If the values are positive, then the shoulders are inclined towards the amputation. On the other hand, negative values indicate that the shoulders are tilted in the opposite direction to the amputation.
angles of lateral curvatures of the spine (LCA, LCNA)	Angles complementary to 180° for the angles located between the lines that connect the tops of the lateral inclinations of the spine with the intersection of the spinal line with the line that connects the spinous processes C7-S1. The values are shown separately for the arcs towards the amputation side and the healthy side, which is the opposite side to the amputation side, so they only take positive values.
Postural Indicators in the Transverse Plane	
pelvic rotation angle (PRA)	The angle between the frontal plane and the line connecting the posterior superior iliac spines. Positive values mean that the pelvis is rotated towards the amputation side. On the other hand, negative values indicate the rotation of the pelvis to the side opposite to the side of the amputation.
trunk rotation angle at the level of the scapula (TRA)	The angle between the frontal plane and the line connecting the lower angles of the shoulder blades. Positive values mean that the torso is twisted towards the amputation side, which means that the scapula on the amputation side is closer to the device than the scapula on the opposite side of the body. On the other hand, negative values mean that the trunk is twisted in the opposite direction to the amputation.

**Table 2:** Moiré topography indices for trials with and without external breast prosthesis (EBS).

Parameters	With EBS								Without EBS							
	M ± SD	Me	Min	Max	Q1	Q3	W	*p*	M ± SD	Me	Min	Max	Q1	Q3	W	*p*
TLA	−11.46 ± 8.49	−9.35	−30.7	−0.3	−13.95	−5.70	0.86	0.00	−10.02 ± 8.59	−7.05	−30.0	0.0	−12.70	−4.25	0.85	0.00
TKA	28.99 ± 13.75	24.35	3.0	62.5	21.25	32.00	0.84	0.00	29.80 ± 14.36	24.95	4.6	66.1	20.25	32.40	0.85	0.00
LLA	23.85 ± 7.99	23.80	3.4	44.0	19.45	28.15	0.98	0.66	24.30 ± 7.28	25.25	5.8	37.0	19.35	30.15	0.97	0.14
CI	5.14 ± 13.45	1.50	−19.1	37.4	−2.90	8.90	0.90	0.00	5.50 ± 14.65	−0.60	−13.1	39.7	−4.80	11.90	0.85	0.00
TIA	−0.08 ± 1.77	−0.05	−3.3	5.0	−1.45	0.95	0.98	0.44	−0.20 ± 1.82	−0.50	−3.8	5.5	−1.25	0.80	0.97	0.23
PIA	−0.62 ± 3.10	−0.70	−10.4	7.8	−2.40	1.55	0.97	0.13	−0.29 ± 2.94	−0.45	−10.4	5.8	−1.50	1.40	0.95	0.02
SLA	0.70 ± 2.55	0.65	−6.2	9.1	−0.95	2.00	0.97	0.18	0.50 ± 2.21	0.75	−4.6	6.8	−1.10	1.50	0.98	0.56
LCA	4.94 ± 2.43	4.40	2.5	13.3	3.40	5.90	0.81	0.00	5.36 ± 2.73	5.00	1.3	14.7	3.50	6.55	0.91	0.01
LCNA	4.66 ± 2.49	4.30	0.5	9.9	3.20	5.20	0.92	0.04	4.50 ± 2.66	3.90	0.9	12.4	2.70	6.00	0.92	0.02
PRA	0.85 ± 5.60	0.50	−11.1	14.3	−2.15	4.10	0.98	0.75	0.66 ± 3.85	0.45	−7.3	9.1	−2.20	2.90	0.99	0.84
TRA	0.20 ± 6.16	0.70	−14.7	10.3	−3.85	4.00	0.96	0.10	0.38 ± 5.62	0.55	−15.0	10.0	−2.90	4.60	0.97	0.24

Abbreviations: TLA—trunk leaning angle, TKA—thoracic kyphosis angle, LLA—lumbar lordosis angle, CI—compensation index, TIA—trunk inclination angle, PIA—pelvic inclination angle, SLA—inclination angle of the shoulder line, LCA—angle of lateral curvature in the spine towards the amputated side, LCNA—angle of lateral curvature in the spine towards the non-amputated side, PRA—pelvic rotation angle, TRA—trunk rotation angle at the level of the scapula, M—mean, SD—standard deviation, Me—median, Q1—lower quartile, Q3—upper quartile, W—Shapiro–Wilk test, *p*—significance value of the test *p* > 0.05.

**Table 3:** Differences in the moiré topography indices for trials with and without external breast prosthesis (EBS).

Parameters	With EBS	Without EBS	*t*	T	Z	*p*
TLA, median (QD)	−9.35 (4.13)	−7.05 (4.23)	−	366.50	2.447	0.014 *
TKA, median (QD)	24.35 (5.38)	24.95 (6.08)	−	554.00	1.229	0.218
LLA, mean ± SD	23.85 ± 7.99	24.30 ± 7.28	−0.481	−	−	0.633
CI, median (QD)	1.50 (5.59)	−0.60 (8.35)	−	622.00	0.610	0.542
TIA, mean ± SD	−0.08 ± 1.77	−0.20 ± 1.82	0.923	−	−	0.361
PIA, mean ± SD	−0.62 ± 3.10	−0.29 ± 2.94	−1.860	−	−	0.069
SLA, mean ± SD	0.70 ± 2.55	0.50 ± 2.21	0.689	−	−	0.494
LCA, median (QD)	4.40 (1.25)	5.00 (1.53)	−	69.50	0.331	0.740
LCNA, median (QD)	4.30 (1.00)	3.90 (1.65)	−	112.50	0.455	0.650
PRA, mean ± SD	0.85 ± 5.60	0.66 ± 3.85	0.271	−	−	0.788
TRA, mean ± SD	0.20 ± 6.16	0.38 ± 5.62	−0.463	−	−	0.645

Abbreviations: TLA—trunk leaning angle, TKA—thoracic kyphosis angle, LLA—lumbar lordosis angle, CI—compensation index, TIA—trunk inclination angle, PIA—pelvic inclination angle, SLA—inclination angle of the shoulder line, LCA—angle of lateral curvature in the spine towards the amputated side, LCNA—angle of lateral curvature in the spine towards the non-amputated side, PRA—pelvic rotation angle, TRA—trunk rotation angle at the level of the scapula, SD—standard deviation, QD—quartile deviation. *t*—Student’s *t*-test, T—Statistical value of the Wilcoxon test, Z—Score-z, *p*—significance value of the test, * Statistic difference.

**Table 4:** Differences between the moiré topography indices in patients after left-sided mastectomy (LSM) and postural indices in patients after right-sided mastectomy (RSM) in trials with and without an external breast prosthesis (EBS).

Parameters	With EBS				Without EBS			
	LSM	RSM	t/U	*p*	LSM	RSM	*t*/U	*p*
TLA, Sum of Ranks	668	710.5	289.5	0.383	631	747.5	252.5	0.120
TKA, Sum of Ranks	714	664	336.0	0.986	755	623	298.0	0.478
LLA, mean ± SD	22.60 ± 7.94	25.20 ± 7.99	−1.177	0.245	23.83 ± 6.16	24.81 ± 8.42	−0.484	0.630
CI, Sum of Ranks	786	592	267.0	0.202	796	582	257.0	0.144
TIA, mean ± SD	0.04 ± 1.76	−0.20 ± 1.80	0.487	0.628	0.06 ± 1.71	−0.48 ± 1.93	1.070	0.290
PIA, mean ± SD	−1.05 ± 3.35	−0.16 ± 2.80	−1.038	0.304	−0.96 ± 3.08	0.43 ± 2.65	−1.742	0.088
SLA, mean ± SD	0.60 ± 2.99	0.80 ± 2.03	−0.280	0.781	0.31 ± 2.62	0.72 ± 1.68	−0.662	0.511
LCA, Sum of Ranks	142	134.5	50.5	0.376	258	270	117.0	0.710
LCNA, Sum of Ranks	158	220.5	79.5	0.614	342	288.5	135.5	0.568
PRA, mean ± SD	2.44 ± 5.33	−0.86 ± 5.48	2.201	0.032 *	2.30 ± 3.10	−1.12 ± 3.85	3.542	0.001 *
TRA, mean ± SD	4.57 ± 3.38	−4.51 ± 4.85	7.878	0.000 *	4.49 ± 3.00	−4.05 ± 4.24	8.423	0.000 *

Abbreviations: TLA—trunk leaning angle, TKA—thoracic kyphosis angle, LLA—lumbar lordosis angle, CI—compensation index, TIA—trunk inclination angle, PIA—pelvic inclination angle, SLA—inclination angle of the shoulder line, LCA—angle of lateral curvature in the spine towards the amputated side, LCNA—angle of lateral curvature in the spine towards the non-amputated side, PRA—pelvic rotation angle, TRA—trunk rotation angle at the level of the scapula, SD—standard deviation, *t*—Student’s *t*-test, U—Mann–Whitney test, *p*—significance value of the test, * Statistic difference.

## Data Availability

Data sets analyzed during the current research are available from the corresponding author upon reasonable request.
